# Heteroatom functionalization of Ti_2_C MXenes for enhanced Li-ion storage performance

**DOI:** 10.1039/d6ra08111h

**Published:** 2026-10-23

**Authors:** Ikram Ud Din, Zeenat Mahal, Amin ur Rashid, Won Chun Oh, Kefayat Ullah

**Affiliations:** a Department of Physics, University of Swat KPK 19120 Pakistan kefsayed@uswat.edu.pk +92-345-920-7686; b Department of Advanced Materials Science & Engineering, Hanseo University Seosan-si Chungnam 31962 Republic of Korea

## Abstract

Surface functionalization provides an effective approach for tailoring the electrochemical properties of MXenes for lithium-ion battery applications. In this study, density functional theory (DFT) calculations were performed to investigate the effect of sulfur (S) and nitrogen (N) terminations on the lithium-storage performance of Ti_2_C MXene. Pristine Ti_2_C together with S-terminated (Ti_2_CS_2_) and N-terminated (Ti_2_CN_2_) monolayers were investigated by evaluating their structural stability, electronic properties, Li adsorption, diffusion behavior, open-circuit voltage and theoretical specific capacity. All three monolayers are energetically stable and retain their metallic character after lithiation, ensuring continuous electronic conductivity during battery operation. Surface functionalization increases the maximum Li atoms from *x*_max_ = 2 in Li_*x*_Ti_2_C to *x*_max_ = 4 in both Li_*x*_Ti_2_CS_2_ and Li_*x*_Ti_2_CN_2_ resulting in theoretical specific capacities of 497.5, 623.7, and 789.7 mAh g^−1^, respectively. The calculated Li-ion diffusion barriers of 0.24, 0.28 and 0.32 eV, together with the favorable open-circuit voltages (OCV) 0.87–0.08 V, 0.81–0.04 V and 0.68–0.02 V for Ti_2_C, Ti_2_CS_2_, and Ti_2_CN_2_ respectively, indicating fast Li-ion transport. Overall, sulfur and nitrogen terminations significantly improve the lithium-storage characteristics of Ti_2_C MXene, with Ti_2_CN_2_ exhibiting the highest theoretical capacity and Ti_2_CS_2_ offering a favorable balance between capacity and Li-ion mobility.

## Abstract 2

Surface functionalization provides an effective approach for tailoring the electrochemical properties of MXenes for lithium-ion battery applications.

## Graphical abstract

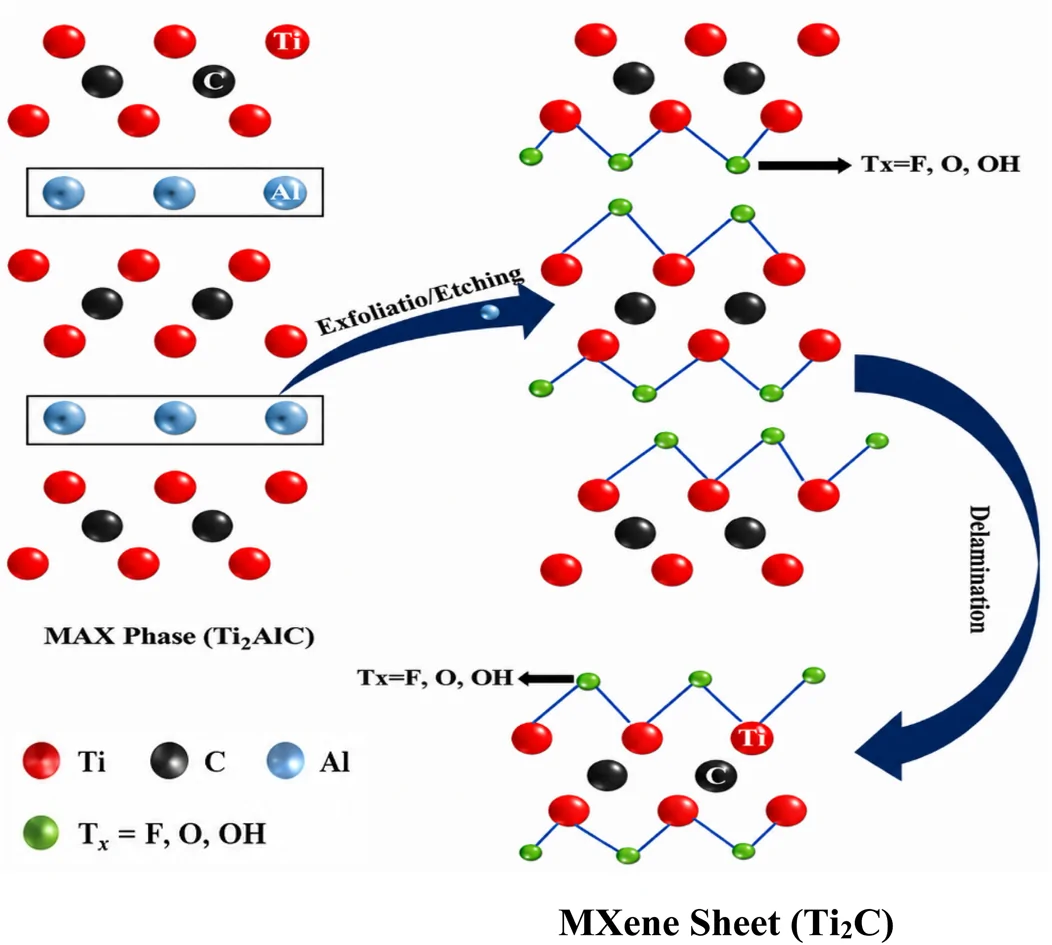

## 1 Introduction

Two-dimensional (2D) materials have gained significant attention because of their exceptional structural, electronic, chemical and mechanical characteristics, which makes them suitable for various technological applications, particularly energy storage applications.^1^ The growing demand for renewable energy and the need to reduce dependence on fossil fuels have increased research interest in efficient energy-storage technologies.^2^ Lithium-ion batteries (LIBs) have become important energy-storage technologies for portable electronics, electric vehicles, and renewable energy systems because of their high energy density and favorable electrochemical performance.^3,4^ However, further improvements in their performance require electrode materials that combine high specific capacity, suitable operating voltage, rapid ion transport and structural stability during repeated charge discharge cycles.^4,5^ Therefore, improvement in these batteries is an urgent need for the grid scale energy storage systems. The battery performance depends significantly on the microstructure and properties of the electrode and electrolyte materials.^6,7^

Since the isolation of graphene in 2004 by Novoselov *et al.*, through mechanical exfoliation,^8–10^ a broad range of 2D materials has been explored, such as transition metal dichalcogenides (TMDs),^11^ hexagonal boron nitride (h–BN),^12^ transition metal oxides,^12^ phosphorene,^13^ borophene^14^ and silicene.^15^ These materials exhibit diverse structural, electronic, and chemical properties that make them promising for various applications, particularly electrochemical energy storage.^16^

Recently, a novel two dimensional (2D) transition metal carbides and nitrides, referred to as MXenes have been synthesized from their layered ternary carbides or nitrides known as MAX phases by selective hydrofluoric acid (HF) etching and subsequent sonication.^17^ The general formula of MAX phase is M_*n*+1_AX_*n*_, in which M denotes an early transition metal, A is generally an element from groups IIIA and IVA, X is either carbon or nitrogen and *n* can be 1, 2, 3 or 4.^18,19^ The M–X bonds in MAX phases are stronger than M–A bonds, allowing selective removal of A layers *via* chemical etching (commonly HF-based) to produce atomically thin MXene sheets.^20,21^ During synthesis, the surfaces of MXenes become functionalized with O, F, and OH groups, forming compositions typically represented as M_n+1_X_*n*_T_*x*_, where T_*x*_ represents surface terminations as shown in Fig. 1. These surface terminations are crucial in modulating MXene electronic and electrochemical properties as well as ion adsorption behavior.^22^ Despite their promising theoretical properties, practical Ti_2_C based electrodes exhibit lower capacities than some idealized theoretical models predict. Naguib *et al.*, reported an experimental capacity of approximately 225 mAh g^−1^ at C/25 for an oxidized Ti_2_C-based material, with capacity decreasing at higher current rates.^23,24^ Moreover, first-principles studies have shown that conventional surface terminations, particularly F and OH can influence Li adsorption and ion migration in MXenes.^25^ These findings highlight the importance of surface termination engineering in tailoring the Li storage properties of Ti_2_C based anodes.

**Fig. 1:**
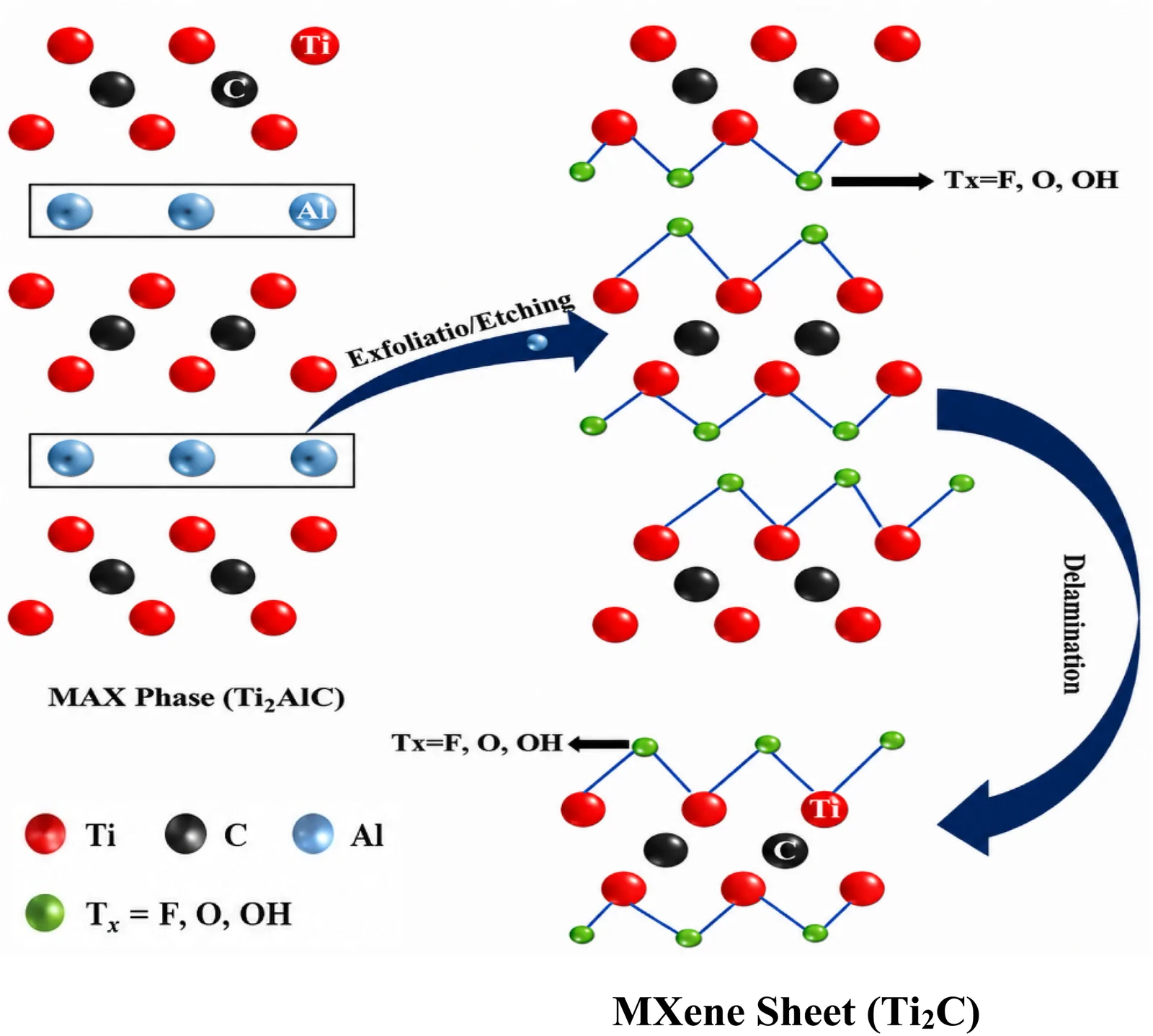
Etching of Ti_2_C MXene from their MAX Phase (Ti_2_AlC) precursor.

After the synthesis of Ti_3_C_2_, almost 30 MXenes have been also experimentally discovered including Ti_2_C, Ti_2_N,^26^ Ti_4_N_3_^27^, V_2_C^28^ Nb_2_C^29^*etc.* and dozens have been theoretically predicted.^30,31^ Recent theoretical investigations have indicated that a wide range of MXenes materials hold significant potential as anode candidates for various metal-ion battery systems particularly LIBs.^32,33^ As shown in Fig. 2, MXenes show unique combination of intrinsic properties, including low open circuit voltage (OCV),^34^ high theoretical specific capacity,^35^ excellent electronic conductivity^36^ and favorable ion diffusion pathways which are essential for high energy storage performance, particularly in batteries and supercapacitors.^37^

**Fig. 2:**
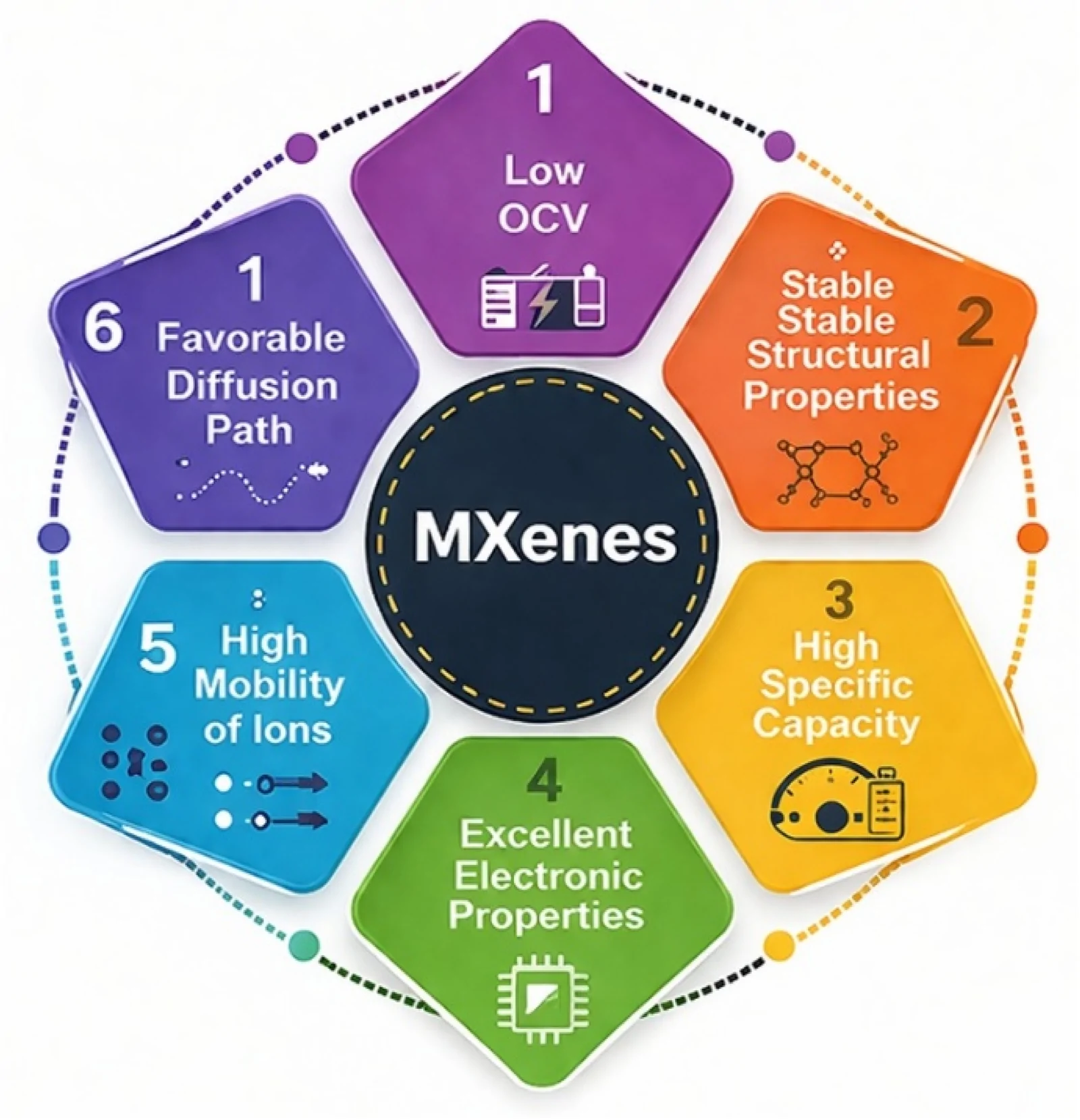
Schematic illustration of key properties of MXenes for electrochemical performance.

Previous theoretical studies have predicted that MXenes such as Sc_2_C,^38^ Ti_2_C,^39^ V_2_C^40^ and Cr_2_C^41^ can deliver gravimetric capacities above 400 mAh g^−1^, which is higher than the theoretical capacity of graphite (372 mAh g^−1^).^42^ Similarly a theoretical capacity of 879 mAh g^−1^ has been predicted for Mn_2_C, indicating excellent lithium storage potential.^43^

Surface functionalization provides an effective route for tuning the electrochemical properties of MXenes, as different termination species can alter Li adsorption, ion diffusion and the availability of favorable Li-storage sites.^44,45^ For example, S- and Se-terminated Ti_3_C_2_ (Ti_3_C_2_S_2_ and Ti_3_C_2_Se_2_) exhibit theoretical Li-storage capacities of 462.6 and 329.3 mAh g^−1^, respectively, together with low Li-diffusion barriers of 0.25 and 0.15 eV.^46^ Similarly, S/O functionalization of V_2_C has been predicted to enhance Li storage, with a theoretical capacity of 729 mAh g^−1^ reported for a mixed-terminated system.^47^ Zr-based MXenes have also shown strong termination-dependent Li-storage behavior. For example, Si-, O-, P-, S- and Se-functionalized Zr_2_C MXenes exhibit different Li adsorption characteristics, with surface termination influencing the strength of Li–surface interactions and Li-storage properties.^47^ In Mo_2_C, surface termination has also been shown to enhance Li-storage capacity. In particular, S-functionalized Mo_2_CS_2_ exhibits a theoretical Li-storage capacity of 400 mAh g^−1^, compared with 263 mAh g^−1^ for pristine Mo_2_C demonstrating the beneficial effect of S termination on Li storage.^48^ Chalcogen-functionalized Nb_2_C has been reported to exhibit high Li-storage capacities, with Nb_2_CSz systems showing values of approximately 409–466 mAh g^−1^ depending on surface termination and lithium concentration.^49^ Experimental studies have further demonstrated that molten-salt reactions can introduce nonconventional MXene surface terminations, including sulfur, selenium, and imido groups.^50^ More recently, N-terminated Ti_3_C_2_N_*x*_ has been experimentally investigated for Li-ion storage.^51^ These studies establish the relevance of termination engineering but do not, by themselves, demonstrate that the idealized Ti_2_CN_2_ structure considered here has been experimentally synthesized. Therefore, a direct comparison of Ti_2_C, Ti_2_CS_2_ and Ti_2_CN_2_ under consistent computational conditions is useful for examining how their surface chemistries influence Li storage capacity, diffusion barriers and open-circuit voltage.

Among various studied MXenes, titanium based MXenes particularly Ti_2_C has gained attention as a 2D material due to its high electrical conductivity, mechanical stability and tunable electronic properties.^52^ Its structure consists of a carbon layer sandwiched between two titanium layers in a hexagonal lattice as shown in Fig. 3(a) and (b).

**Fig. 3:**
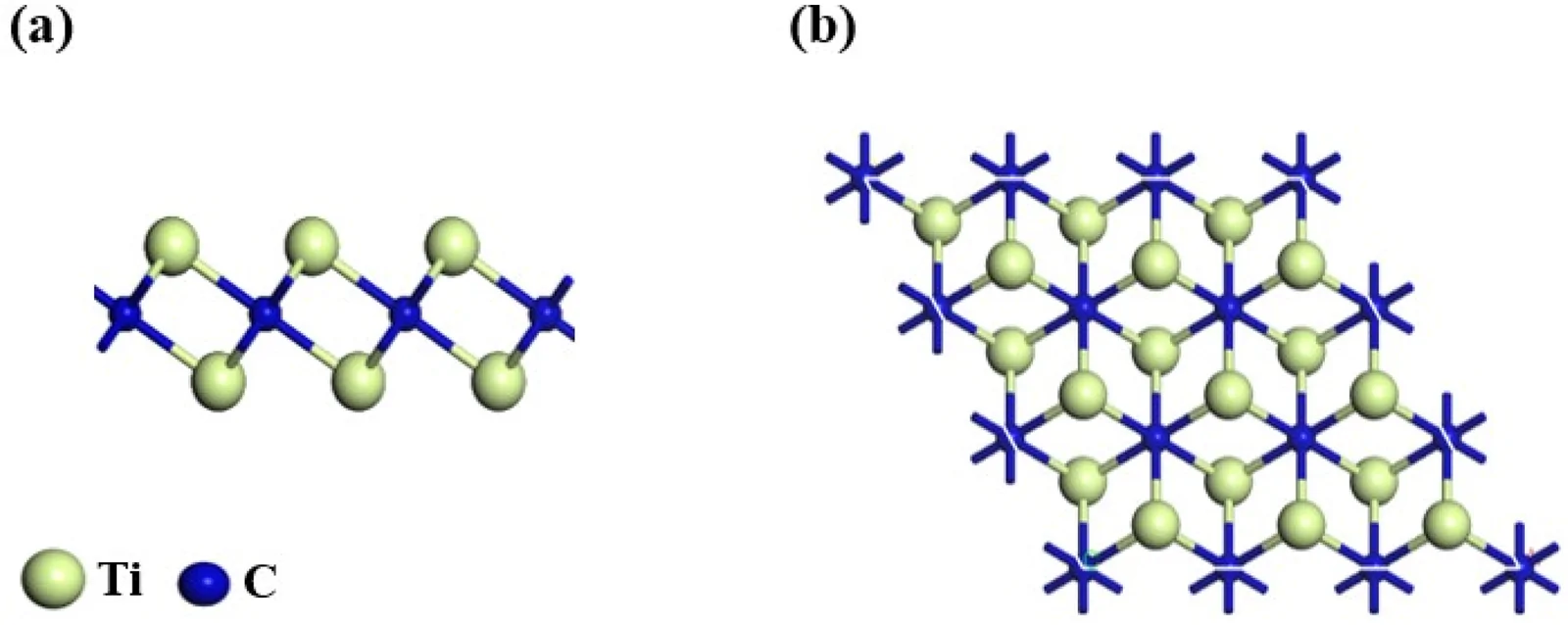
Optimized atomic configuration of pristine Ti_2_C (a) side and (b) top view.

Computational studies predict that Ti_2_C offers relatively low diffusion barriers for alkali and multivalent ions making it an attractive candidate exhibits strong performance in lithium-ion batteries while also being suitable for sodium and magnesium-ion systems.^53,54^ Theoretical studies further suggests that electronic behavior of Ti_2_C can be adjusted through surface functionalization like –O, –OH, F *etc.* and it exhibits strong affinity for lithium ions, making it a suitable anode material for lithium-ion batteries.^55^ To provide a quantitative comparison with previously reported Ti_2_C-based MXenes, the Li-storage capacities and Li-diffusion barriers of pristine and surface functionalized Ti_2_C systems are summarized in Table 1.

**Table 1:** Comparison of Li-storage capacity and Li-diffusion barriers of Ti_2_C-based MXenes

Ti-based MXenes	Surface functional groups	Li-storage capacity (mAh g^−1^)	Li-diffusion barrier (eV)	References
Ti_2_C	Pristine	497.5	0.24	This work
Ti_2_CS_2_	S	623.7	0.28	This work
Ti_2_CN_2_	N	789.7	0.32	This work
Ti_2_CO_2_	O	348.9	0.27	56
Ti_2_CF_2_	F	280	0.34	57 and 58
Ti_2_C(OH)_2_	OH	344.4	—	59
Ti_2_CCl_2_	Cl	262	0.27	58 and 60

Furthermore, the theoretical Li-storage capacity depends on the number of Li atoms accommodated in the first adsorption layer, located closest to the MXene surface, and in additional layers positioned farther from the surface. The contribution of the second Li layer should therefore be considered in relation to the stability of the fully lithiated configuration, which is further examined here using AIMD simulations.

In this study, first principles calculations based on DFT were performed to explore Ti_2_C MXenes as an electrode material for LIBs along with their functionalized forms. Differences in the electronegativity and orbital character of S and N may modify the local bonding environment and electronic structure of the Ti-based surfaces thereby influencing Li adsorption energetics, ion-migration barriers and OCV.^61^ To examine these effects, we compare the structural and electronic properties, Li adsorption configurations and energies, theoretical storage capacities, diffusion barriers and OCV profiles of the three systems. The stability of pristine Ti_2_C and its functionalized forms are further confirmed through phonon dispersion and AIMD simulations. Additionally, density of states (DOS) calculations reveals that both pristine and functionalized Ti_2_C MXenes exhibit metallic character and excellent electronic conductivity. This comparative investigation aims to clarify the trade-offs among theoretical Li-storage capacity, ion mobility and operating voltage associated with S and N termination.

Overall, this study highlights the significant potential of titanium-based MXenes, particularly Ti_2_CT_*x*_ as promising electrode materials for LIBs and provides valuable insights for future exploration of other metal-ion battery applications.

## 2 Computational methods

In this work, first principles calculations were carried out using CASTEP module within the Materials Studio software.^62^ The interaction of valence electrons with ionic cores was modeled using ultrasoft pseudopotentials, while the exchange correlation effects was evaluated within the framework of generalized gradient approximation (GGA) using the Perdew–Burke–Ernzerhof (PBE) functional.^63,64^ The Grimme DFT-D dispersion correction, as implemented in CASTEP, was enabled to account for dispersive interactions. A 2 × 2 × 1 supercell was constructed for geometry optimization and electronic structure calculations, with the Brillouin zone integration using an 8 × 8 × 1 Monkhorst–Pack *k*-point grid.^65^ A vacuum layer of 20 Å was introduced along the *z*-direction to ensure sufficient separation between periodic images. Based on convergence tests, cutoff energy for plane-wave basis was fixed at 550 eV. The convergence criteria for self-consistent field (SCF) calculations were set to 1.0 × 10^−5^ eV per atom for total energy, 0.03 eV Å^−1^ for maximum force, 0.001 Å for maximum atomic displacement and 0.05 GPa for maximum residual stress.

Phonon dispersion calculations were performed using a 2 × 2 × 1 supercell within the framework of density functional perturbation theory (DFPT). Norm-conserving pseudopotentials were used for the phonon calculations. Thermal stability was examined through AIMD simulations at 500 K for 5 ps for pristine and 8 ps for functionalized Ti_2_C using the NVT ensemble with a time step of 1 fs to ensure numerical accuracy. A 2 × 2 × 1 supercell containing 17 atoms for Ti_2_C and 25 atoms each for Ti_2_CS_2_ and Ti_2_CN_2_ was used for the AIMD simulations. The adsorption behavior of Li on Ti_2_C, Ti_2_CS_2_ and Ti_2_CN_2_ monolayers was investigated using a 3 × 3 × 1 supercell. To calculate the Li adsorption energies and open-circuit voltages (OCVs), bulk metallic Li was used as the reference state. The reference energy per Li atom was obtained from periodic DFT calculations by optimizing the body-centered cubic (bcc) structure of bulk Li and dividing the converged total energy by the number of Li atoms in the unit cell. The use of bulk bcc Li as the reference state is consistent with previous first-principles studies of MXene-based anodes.^48,56^

## 3 Results and discussion

### 3.1 Structural properties of Ti_2_C and functionalized Ti_2_CT_*x*_ (T_*x*_ = S and N)

The results obtained indicate that the ground-state structure of bare Ti_2_C MXenes is found to be trigonal, belonging to the *P*3̄*m*1 (No.164) space group. As shown in Fig. 3(a) and (b), both top and side views of a single Ti_2_C layer consist of three alternating sheets arranged in the Ti(1)–C–Ti(2) sequence.

This structure can be visualized as a “sandwich” where a carbon layer is positioned between two titanium layers. The lattice parameters were found to be *a* = *b* = 3.012 Å, with a Ti–C bond length of 2.098 Å, values that are in close agreement with previously reported results.^66^

Several functional groups including sulfur (S) and nitrogen (N) were attached to the optimized Ti_2_C structure. To investigate the most favorable site, three different possible sites were studied as illustrated in Fig. 4(a)–(c). Site I which is positioned with carbon atoms on both side of Ti_2_C, site II is situated at the hollow centers enclosed by the surrounding carbon atoms on both sides and site III corresponds to the termination positions above the Ti (2) atoms on one side and below the C atoms on the opposite side of Ti_2_C monolayer.

**Fig. 4:**
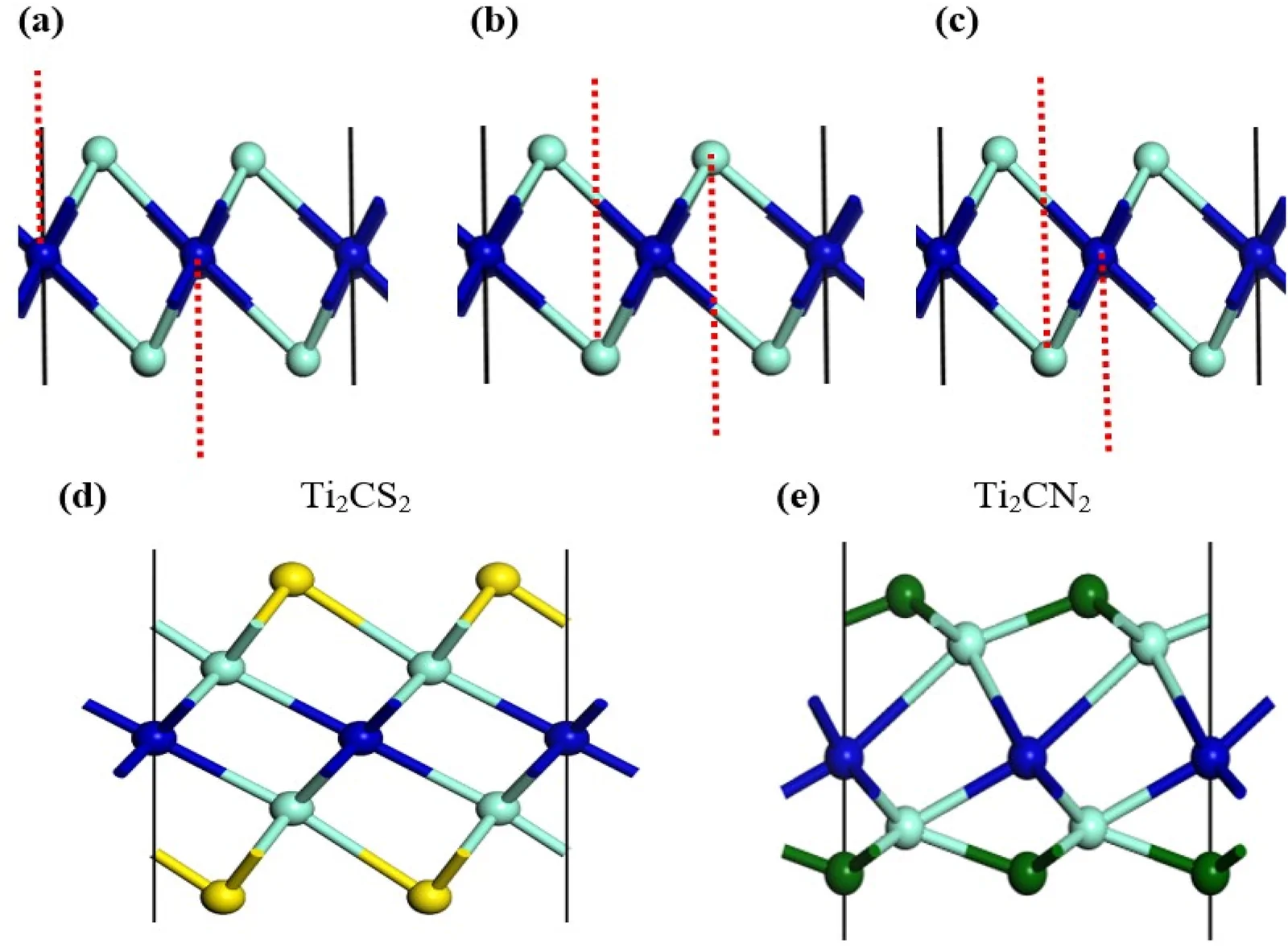
Three possible surface termination configurations for S- and N-terminated Ti_2_C (a) site I (b) site II (c) site III. Side views the optimized atomic structures of the energetically most stable functionalized (d) Ti_2_CS_2_ (e) Ti_2_CN_2_.

After structural optimization, the lattice parameters and formation energies of both pristine Ti_2_C and functionalized Ti_2_CT_2_ (T = S and N) are summarized in Table 2. Here *a* represents the lattice constant of the Ti-based MXenes structures, while *E*_f_ denotes the formation energy per atom of the pristine and functionalized MXenes.

**Table 2:** Optimized lattice parameters including Ti–C bond lengths, Ti–T bond lengths where T = S, F, (OH) and formation energies *E*_f_ (per atom)

Compound	*a*, *b* (Å)	Ti–C (Å)	Ti–T (Å)	*E*_f_ (eV per atom)
Ti_2_C	3.012	2.09	—	−7.91

**Ti**_**2**_ **CS**_**2**_				
Site I	3.23	2.18	2.43	−7.36
Site II	3.22	2.21	2.38	−7.56
Site III	3.19	2.19	2.07	−6.83

**Ti**_**2**_ **CN**_**2**_				
Site I	3.04	2.21	2.01	−8.77
Site II	3.10	2.28	1.96	−8.50
Site III	3.21	2.18	2.06	−8.87

The results obtained after geometry optimization shows that the most favorable site for Ti_2_CS_2_ MXenes corresponds to Site II, while Ti_2_CN_2_ because of the stronger Ti–N bonds and lowers the total energy, preferred site III as shown in Fig. 4(d) and (e) respectively. The results are consistent with the previous reported work.^67–69^

To find the structural stability of both Ti_2_C and Ti_2_CT_2_, the formation energy per atom was calculated using eqn (1) and (2) respectively.^70,71^1
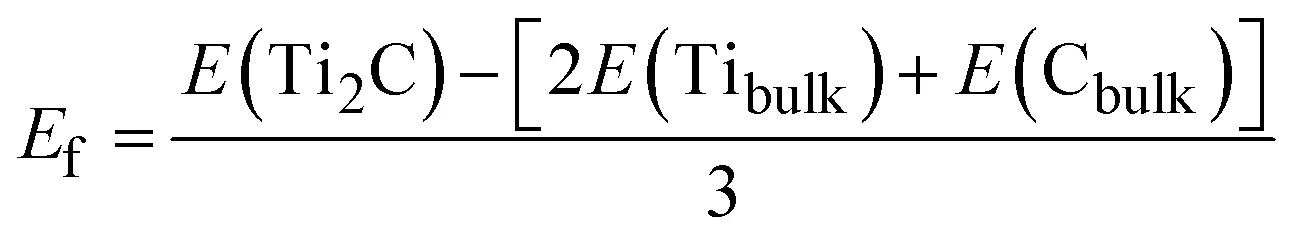
2
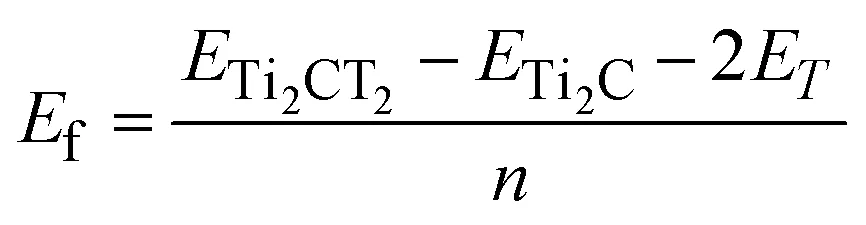
where the terms, *E*(Ti_2_C) refers to the total energy of the pristine Ti_2_C monolayer, whereas *E*(Ti_bulk_)and *E*(C_bulk_) are the energies per atom of the corresponding bulk elemental phases of Ti and C respectively. For the functionalized systems, Ti_2_CT_2_ is the total energy of functionalized Ti_2_C based MXenes, *E*(Ti_2_C) represents the energy of the pristine monolayer, and *E*(T) is the energy of the elemental phase of the termination species. The results obtained shows that all Ti_2_C and functionalized Ti_2_CS_2_ and Ti_2_CN_2_ have sable structures.

### 3.2 Stability of Ti_2_C and functionalized Ti_2_CS_2_ and Ti_2_CN_2_

#### 3.2.1 Thermal stability

The thermal stability Ti_2_C, Ti_2_CS_2_ and Ti_2_CN_2_ was examined using *ab initio* molecular dynamics (AIMD)^72^ simulations at 500K over a time scale of 5 ps. As shown in Fig. 5(a)–(c), the free energies of Ti_2_C, Ti_2_CS_2_ and Ti_2_CN_2_ stabilizes gradually around −257, −540 and −408 eV respectively during the time 5 ps at 500 K. Minor distortions observed in the final configuration are attributed to thermal vibrations and do not affect the overall stability of the system. These results confirm the excellent thermal robustness of Ti_2_C, Ti_2_CS_2_ and Ti_2_CN_2_ suggesting its suitability for high-temperature electrochemical applications.

**Fig. 5:**
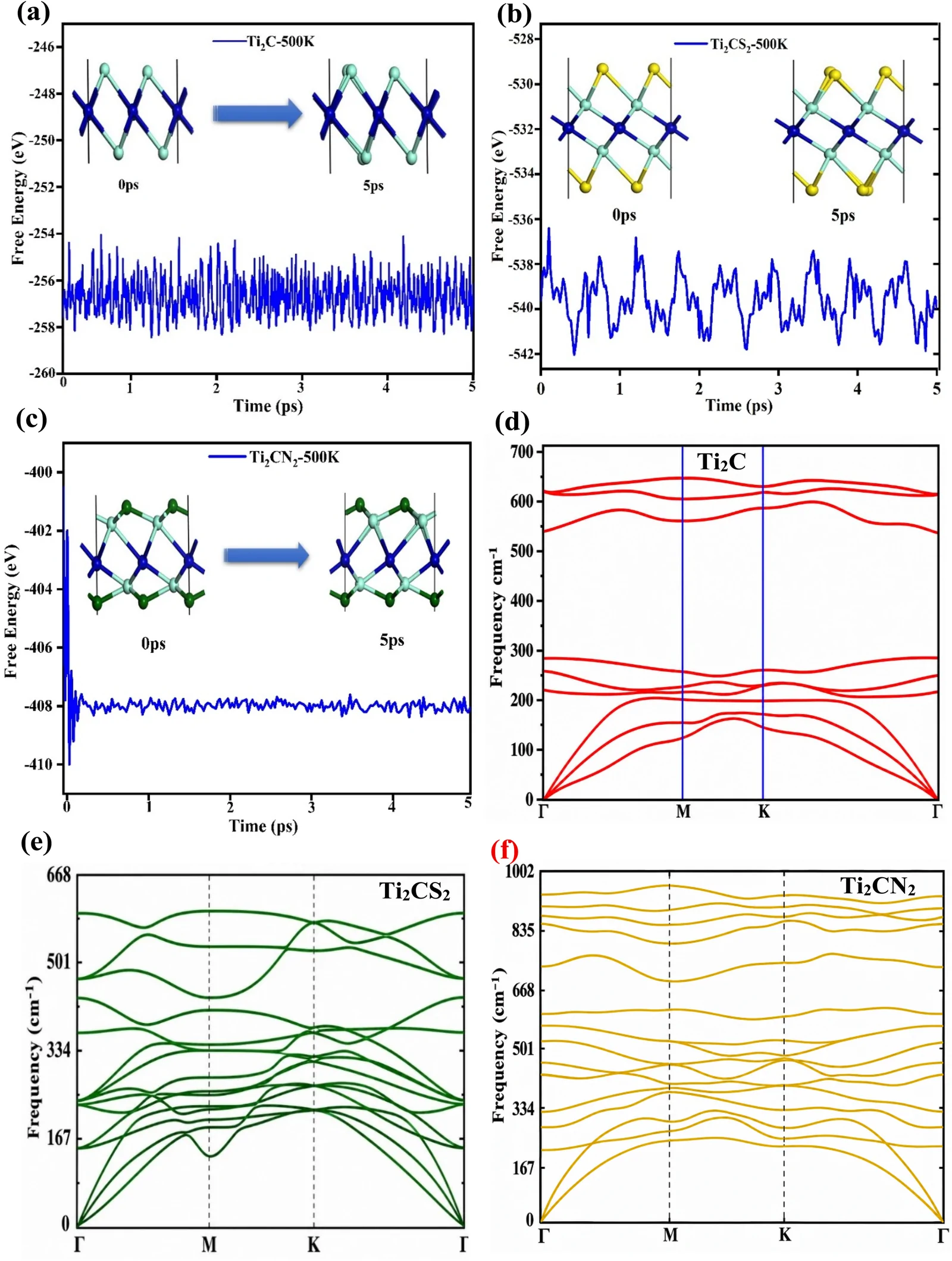
Variation of free energy and structure in AIMD simulation at 500 K (a) Ti_2_C and (b) Ti_2_CS_2_ (c) Ti_2_CN_2_. Phonon dispersion curves for (d) Ti_2_C (e) Ti_2_CS_2_ (f) Ti_2_CN_2_.

#### 3.2.2 Dynamic stability

Phonon dispersion calculations were performed to confirm the dynamic stability of Ti_2_C and functionalized Ti_2_CS_2_ and Ti_2_CN_2_ MXenes as shown in Fig. 5(d)–(f). The results show that there are no imaginary frequencies in the whole Brillion zone providing clear evidence of the dynamic stability of Ti_2_C MXenes.

The combined confirmation of both dynamic and thermal stability reinforces the potential of Ti_2_C and functionalized Ti_2_CS_2_ and Ti_2_CN_2_ as a reliable electrode material particularly for applications operating in high-temperature electrochemical environments.

### 3.3 Electronic properties of Ti_2_C monolayer and Ti_2_CT_*x*_ (T_*x*_ = S and N)

The electronic properties of anode materials play a vital role in determining cycling performance, rate capability and conductivity of a rechargeable metal-ion batteries.^73^ To evaluate the intrinsic electronic behavior of pristine Ti_2_C and functionalized Ti_2_C, first-principles calculations of its band structure as well as total (TDOS) and partial density of states (PDOS) were performed. For Ti_2_C, Ti_2_CS_2_ and Ti_2_CN_2_ the calculated band structure shows multiple bands crossing the Fermi level as shown in Fig. 6(a)–(c), confirming the intrinsic metallic nature of the pristine Ti_2_C and functionalized Ti_2_C. This metallic behavior confirms excellent electrical conductivity promoting efficient charge transport in lithium-ion batteries. The total density of states (TDOS) and partial density of states of pristine Ti_2_C and Ti_2_CT_*x*_ (T_*x*_ = S and N) was evaluated and the results are presented in Fig. 6(d)–(f). The absence of an energy band gap near the Fermi level confirms that Ti_2_C and Ti_2_CT_*x*_ (T_*x*_ = S and N), exhibits metallic behavior, which is favorable for high electronic conductivity and the partial density of states study reveals that the electronic states close to the Fermi level are mostly dominated by the contributions of Ti-3d orbitals for both pristine and functionalized Ti_2_C, facilitating rapid electron transfer during electrochemical processes. These characteristics indicate that titanium based MXenes particularly Ti_2_C possesses abundant electronic states capable of facilitating efficient electron transport highlighting its potential as a high-performance conductive material.

**Fig. 6:**
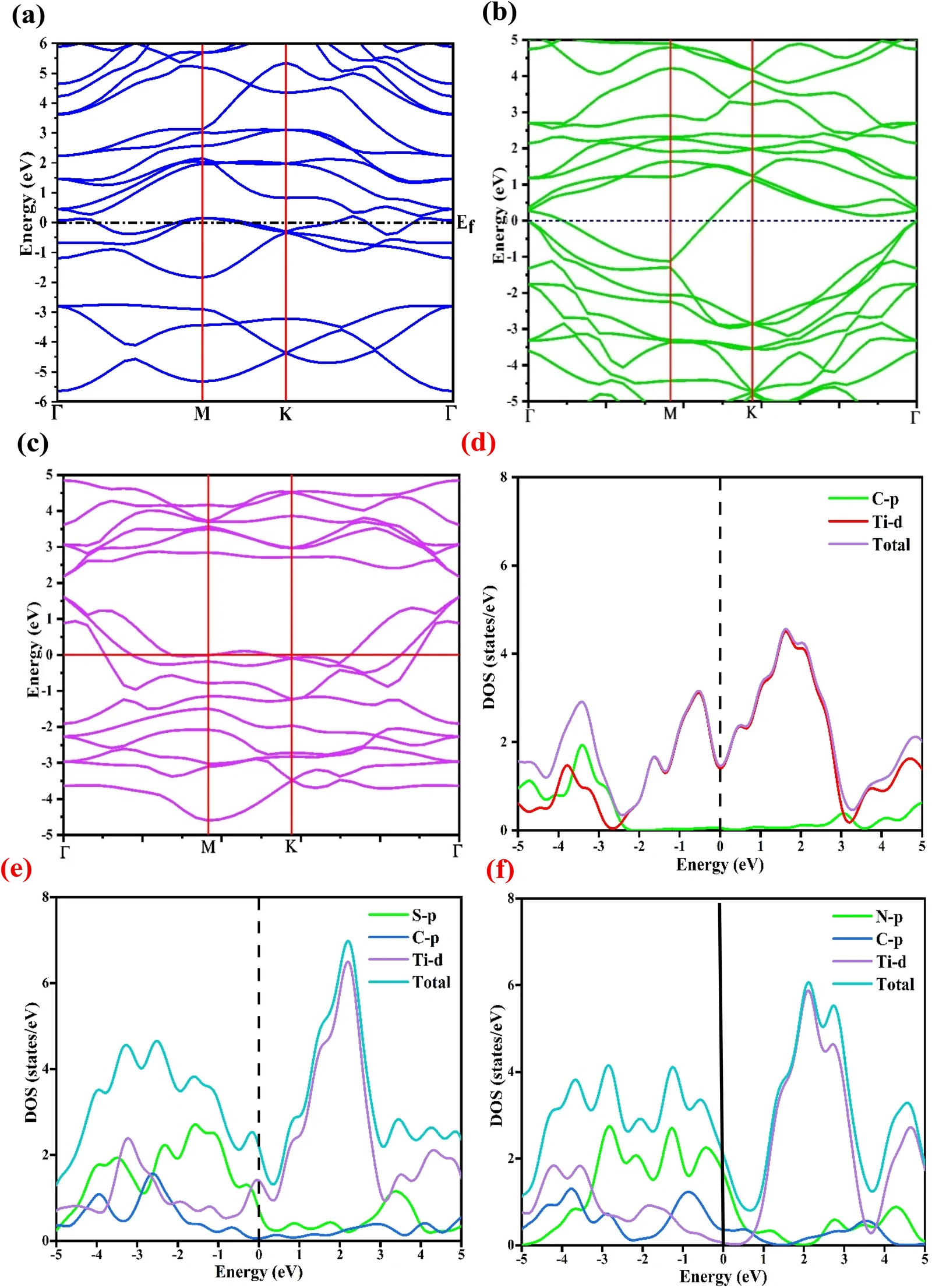
Band structures of (a) Ti_2_C (b) Ti_2_CS_2_ (c) Ti_2_CN_2_ and density of states (d) Ti_2_C (e) Ti_2_CS_2_ (f) Ti_2_CN_2_.

### 3.4 Lithium storage performance of bare Ti_2_C and functionalized Ti_2_CS_2_ and Ti_2_CN_2_ monolayer

To explore the potential applications of Ti_2_C, S-terminated Ti_2_CS_2_ and N-terminated Ti_2_CN_2_ as an anode material, we calculate different parameters including adsorption energies, diffusion barrier, theoretical capacity and open circuit voltage.^74^ Adsorption energy plays a vital role in determining suitable adsorption sites for Li-ion storage. The 3 × 3 × 1 supercell was built to study the most favorable adsorption sites for single Li-ions on bare and functionalized Ti_2_C monolayer. For Ti_2_C and Ti_2_CT_*x*_ (T_*x*_ = S and N), three possible sites are considered as shown in Fig. 7(a) and (b) respectively, that is α (above C atom), β (above Ti(2) atom) and γ (top Ti (1) atom).

**Fig. 7:**
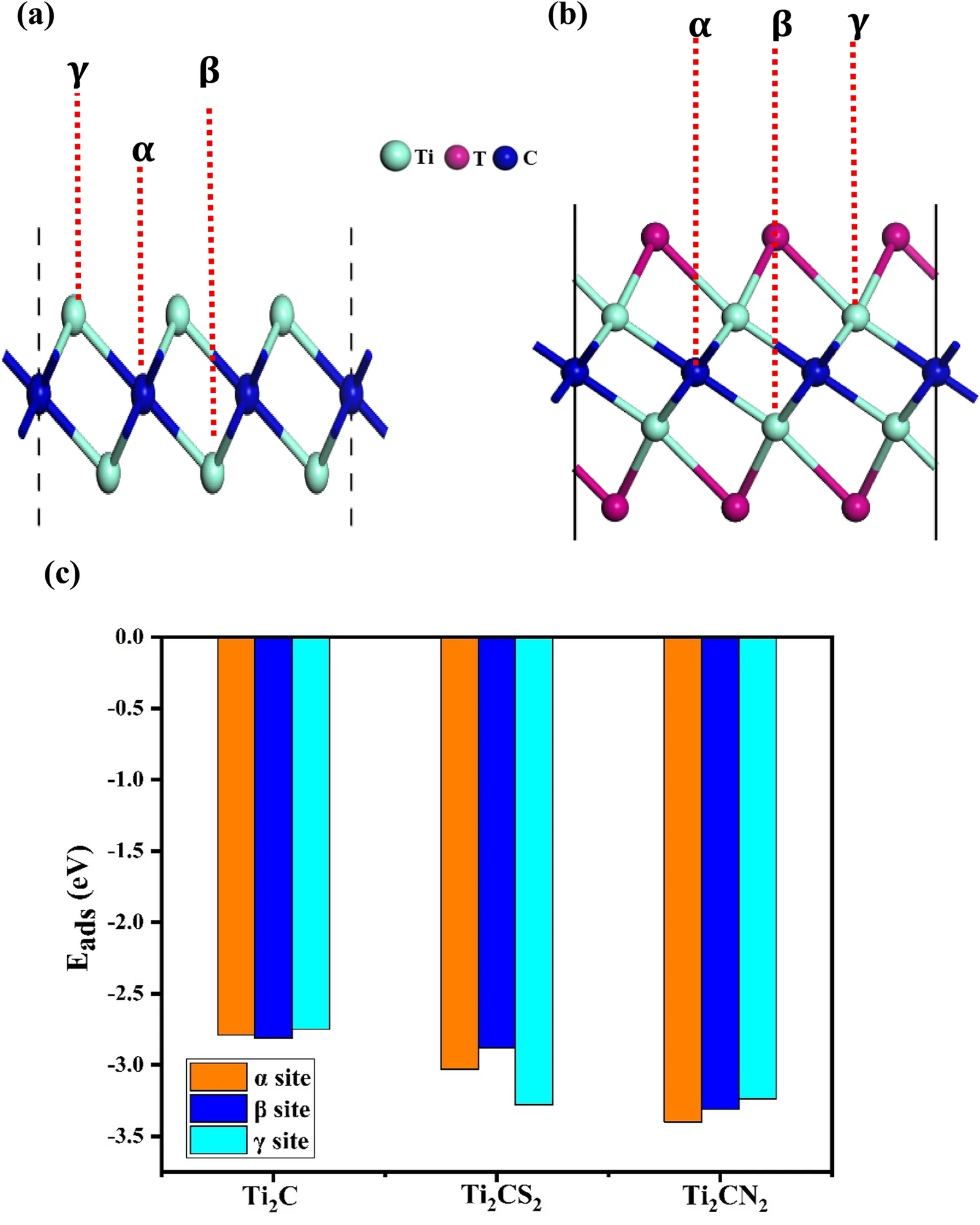
Adsorption sites for Li on (a) Ti_2_C (b) Ti_2_CT_*x*_ (T_*x*_ = S and N) (c) adsorption energies of Li atoms at the α, β, and γ adsorption sites on pristine Ti_2_C, Ti_2_CS_2_ and Ti_2_CN_2_.

We use the formula^75^ to identify the most stable site for ion adsorption.3
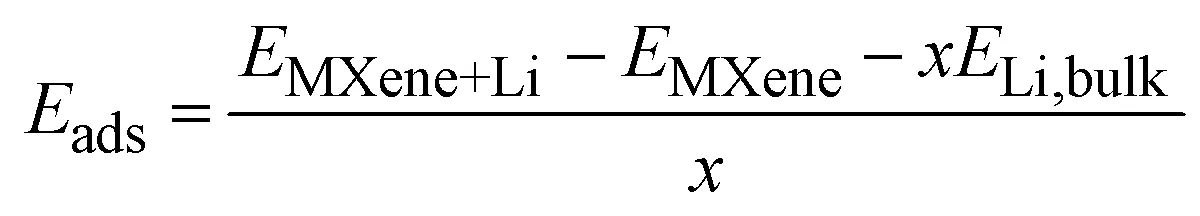
where *E*_ads_ represents the adsorption energy, while *E*_MXene+Li_ and *E*_MXene_ denotes the total energy of Lithiated and pristine MXenes respectively. *E*_Li_ represents energy per atom of the bulk lithium and *x* is the number of adsorbed Li-ion. A more negative value of *E*_ads_ corresponds to stronger adsorption,^76^ indicating that the metal atoms are likely to bind spontaneously on MXene monolayer which hinders the formation of clusters.^77^ The obtained values of adsorption energies for single Li atom at various sites indicates that surface functionalization significantly influences the preferred adsorption position of Li. For Ti_2_C, β site has the lowest adsorption energy among all adsorption sites indicating that it is the most favorable site for Li^+1^ adsorption. For S-terminated Ti_2_CS_2_, γ site and for N-terminated Ti_2_CN_2_, α site is the most favorable site as shown in Fig. 7(b) which is consistent with the previous reported work.^69,78^

The preferred Li adsorption site changes after surface functionalization because the S and N atoms alter the local bonding environment of the Ti_2_C surface. These changes affect the interaction between Li and the neighboring atoms, leading to different adsorption energies and stable adsorption sites.^79,80^ The comparison of adsorption energies is presented in Fig. 7(c).

The most stable adsorption energies of Li on Ti_2_C, Ti_2_CS_2_ and Ti_2_CN_2_ are −2.81, −3.28 and −3.40 eV respectively. These results indicate that N functionalization provides the strongest Li adsorption, followed by S functionalization, while pristine Ti_2_C exhibits the weakest interaction. Therefore, the stability of Li adsorption follows the order: Ti_2_CN_2_ > Ti_2_CS_2_ > Ti_2_C. The adsorption energy values for different sites are presented in Table 3, which are in close agreement with the previously reported work.^39,54,81,82^

**Table 3:** Calculated adsorption energies (*E*_ads_), Li–Ti bond lengths and nearest surface bond lengths for Li adsorbed at the α, β and γ sites on pristine Ti_2_C, Ti_2_CS_2_ and Ti_2_CN_2_ monolayers

MXenes	Adsorption site	*E*_ads_ (eV)	*d*_Li–Ti_ (Å)	*d*_Ti–X_ (Å)
Ti_2_C	α	−2.79	2.21	2.12 (Ti–C)
	β	−2.81	2.14	2.10 (Ti–C)
	γ	−2.75	2.25	2.11 (Ti–C)
Ti_2_CS_2_	α	−3.03	2.46	2.43 (Ti–S)
	β	−2.88	2.50	2.44 (Ti–S)
	γ	−3.28	2.38	2.43 (Ti–S)
Ti_2_CN_2_	α	−3.40	2.04	1.96 (Ti–N)
	β	−3.31	2.08	1.97 (Ti–N)
	γ	−3.24	2.12	1.98 (Ti–N)

To further investigate charge redistribution upon Li adsorption, Hirshfeld charge analysis was performed, and the average atomic charges are summarized in Table 4. For Ti_2_CS_2_, the average Hirshfeld charges of Li atoms in the first and second adsorption layers are +0.76 and +0.80 |e|, respectively, whereas the corresponding values for Ti_2_CN_2_ are +0.86 and +0.78 |e|. The positive charges indicate electron donation from Li atoms to the underlying substrates, suggesting that charge transfer persists beyond the first adsorption layer.

**Table 4:** Average Hirshfeld charges (in electrons) for Li-adsorbed Ti_2_C, Ti_2_CS_2_, and Ti_2_CN_2_

MXenes	Atom	Charge (*e*)
Ti_2_C + 18Li	Ti_1_	−0.15
	Ti_2_	−0.12
	C	−0.45
	Li (first layer)	+0.83
Ti_2_CS_2_ + 36Li	Ti_1_	−0.10
	Ti_2_	−0.08
	C	−0.35
	S	−0.30
	Li (first layer)	+0.76
	Li (second layer)	+0.80
Ti_2_CN_2_ + 36Li	Ti_1_	−0.12
	Ti_2_	−0.09
	C	−0.30
	N	−0.40
	Li (first layer)	+0.86
	Li (second layer)	+0.78

Furthermore, Hirshfeld charge analysis shows that surface termination influences charge transfer during lithiation. In pristine Ti_2_C, the average charge on Li atoms in the first adsorption layer is +0.83 |e|. For Ti_2_CS_2_, the Li charge increases from +0.76 |e| in the first layer to +0.80 |e| in the second layer. In contrast, Ti_2_CN_2_ shows a decrease from +0.86 |e| to +0.78 |e| across the corresponding layers. These results indicate that the two terminations affect the charge-transfer behavior of Li atoms in different adsorption environments. The calculated charges on *S* (−0.30 |e|) and *N* (−0.40 |e|) also indicate electron accumulation on the termination atoms with N exhibiting the more negative charge.

After Li adsorption, it is important to investigate the electronic properties of the resulting Lithiated systems because good electronic conductivity is beneficial for charge transport during battery operation.^83^ To understand the electronic behavior, the total density of states (TDOS) of Lithiated Ti_2_C, Ti_2_CS_2_, and Ti_2_CN_2_ are presented in Fig. 8. The calculated DOS profiles show that the electronic states cross the Fermi level (*E*_F_), indicating the metallic character of these monolayers is retained in the Lithiated system.

**Fig. 8:**
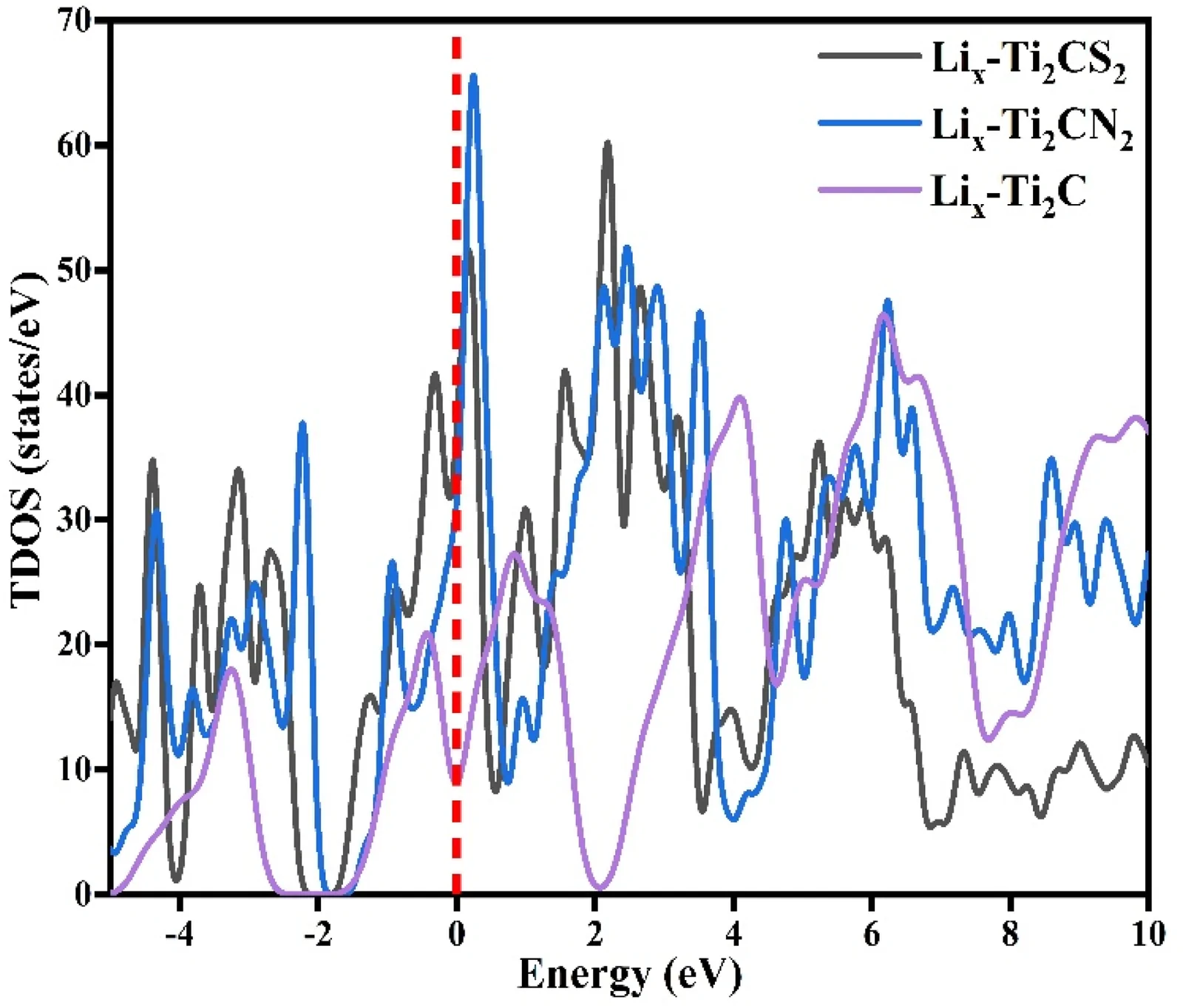
Total density of states (TDOS) of pristine and Li-adsorbed Ti_2_C, Ti_2_CS_2_, and Ti_2_CN_2_ monolayers. The vertical red dashed line indicates the Fermi level (*E*_F_ = 0 eV).

#### 3.4.1 Diffusion of Li-ion on bare Ti_2_C and Ti_2_CT_*x*_ (T_*x*_ = S and N) monolayer

The rate capability of Li-ion batteries is strongly controlled by how easily metal ions can move across the electrode surface.^84^ In this work, the migration behavior of Li on pristine Ti_2_C and Ti_2_CT_*x*_ (T_*x*_ = S and N) monolayers were examined. Three distinct Li-diffusion pathways were examined using the LST/QST method implemented in CASTEP through materials studio as shown in Fig. 9(a–c). For Ti_2_C, Path-I show the diffusion route directly from the top site of the Ti (2) atom to the top-site of a neighboring Ti (2) atom. Path-II shows that the Li diffuses from the top-site of the bottom Ti atom to an intermediate the top-site of the middle C atom, and then moves to the stable top-site of the neighboring bottom Ti (2) atom. follows a trajectory from the top site of a Ti(2) atom through an intermediate site above the top Ti (1) atom, ending at the adjacent Ti (2) top site. In Ti_2_CS_2_, Path I connects directly the adsorption sites above neighboring upper-layer Ti (1) atoms. Path II passes through an intermediate site above C, whereas Path III proceeds through an intermediate site above S; both routes connect neighboring Ti (1) related adsorption sites. In Ti_2_CN_2_, Path I connects neighboring C related adsorption sites directly. Path II proceeds through an intermediate site above Ti (1), while Path III passes through an intermediate site above N before reaching a neighboring C related site.

**Fig. 9:**
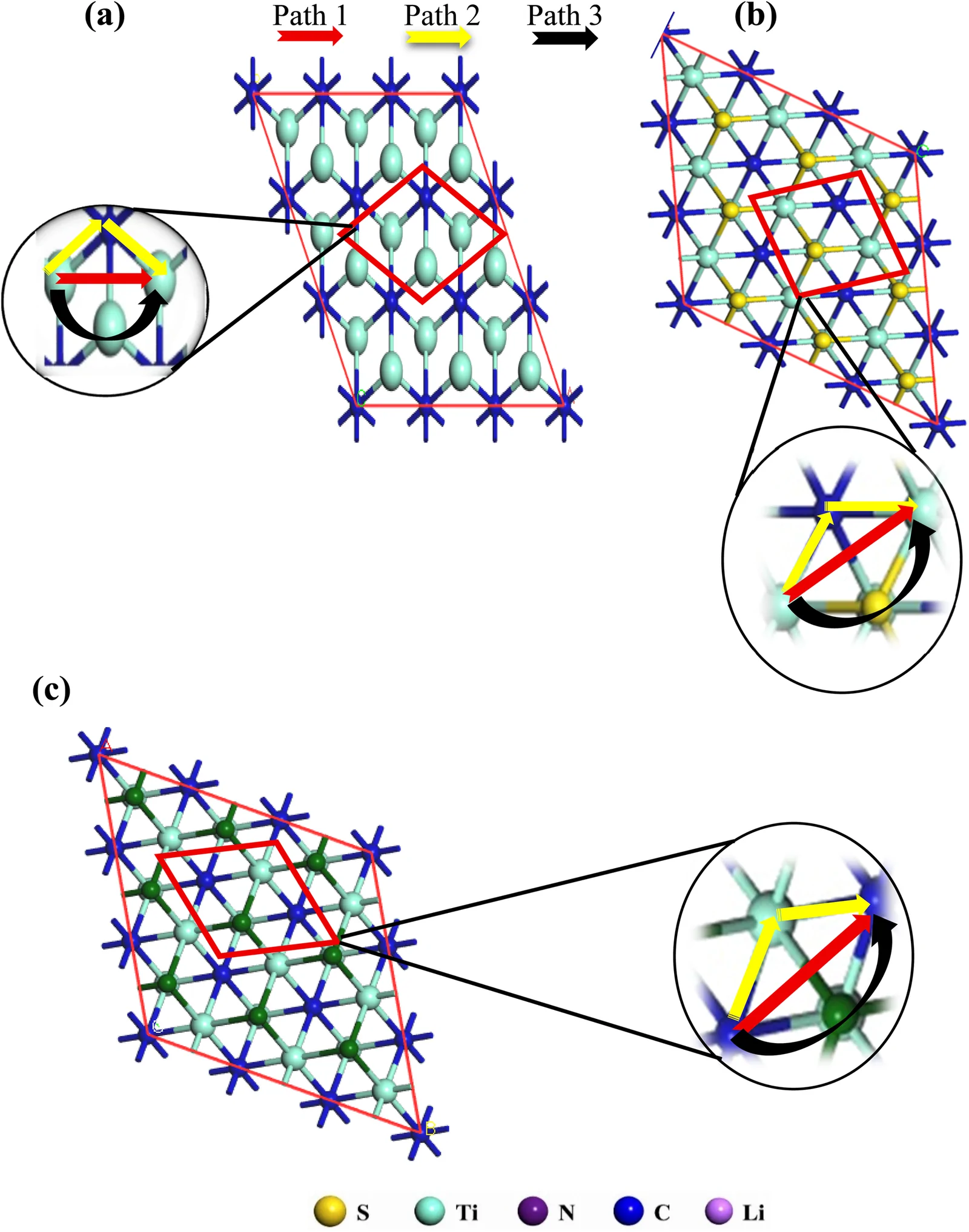
Schematic illustrations of the three possible Li-ion diffusion pathways in (a) Ti_2_C, (b) Ti_2_CS_2_ and (c) Ti_2_CN_2_.

The most stable adsorption site of Li on Ti_2_C and Ti_2_CT_*x*_ (T_*x*_ = S and N) are identified using eqn (3). The calculated Li-ion diffusion barriers are 0.24 eV for Ti_2_C (Path II), 0.28 eV for Ti_2_CS_2_ (Path II) and 0.32 eV for Ti_2_CN_2_ (Path II), which are close to early reported work.^39,57^ Among the three systems, pristine Ti_2_C exhibits the lowest migration barrier, indicating the most favorable Li-ion transport while S and N functionalization slightly increases the diffusion barrier because of the stronger interaction between Li and the terminated surfaces. The energy *versus* relative diffusion coordinates is depicted in Fig. 10(a) showing that the diffusion barrier values for all these MXenes are considerably lower than that of conventional graphite electrodes (0.45–1.2 eV)^85^ and also smaller than several reported values for functionalized MXenes, including Ti_3_C_2_F_2_ (0.36 eV) and Ti_3_C_2_(OH)_2_ (0.90 eV).^53,86^ The relatively low diffusion barrier obtained for all these MXenes reflects easier Li migration on the surface, indicating improved ion transport characteristics and supporting its suitability for high-rate energy storage applications.^37^

**Fig. 10:**
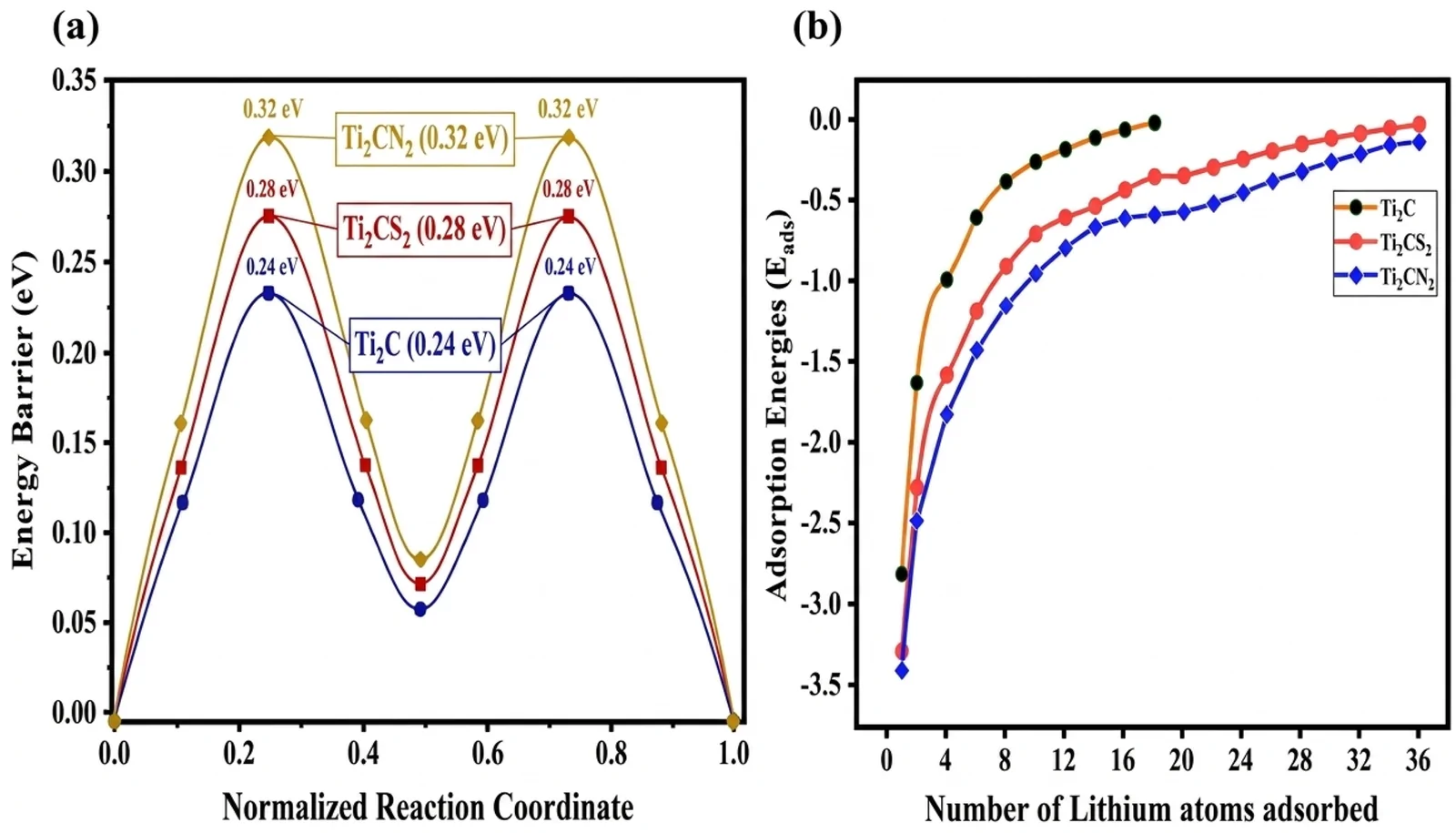
(a) Diffusion barrier profile for single Li atom adsorbed on Ti_2_C, Ti_2_CS_2_ and Ti_2_CN_2_ (b) number of lithium atom adsorbed and their corresponding adsorption energies.

To evaluate the Li storage performance of Ti_2_C, Ti_2_CS_2_ and Ti_2_CN_2_ monolayers, Li atoms were gradually adsorbed on both sides of the 3 × 3 × 1 supercells at the most stable adsorption sites determined from the single Li adsorption calculations. Similar site-selection and sequential Li-adsorption approaches have been employed in previous first-principles investigations of MXene-based anodes.^87,88^ Previous computational studies on MXene electrodes, including Ti_3_C_2_, Nb_2_C, V_2_C, Cr_2_C and Mo_2_CS_2_ materials, have demonstrated that multilayer Li adsorption is an effective approach for enhancing charge-storage capacity.^89^ The lithiation process can be described by using eqn 4 ,

4 *Y* + *x*Li^+^ + *x*ē → Li_*x*_|*Y*

where *Y* represents pristine, S- or N-terminated surface. The average adsorption energy is calculated using eqn (5),^69,90^5
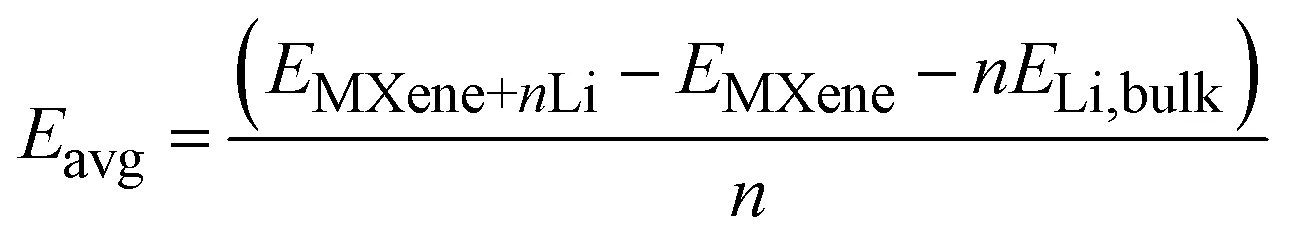
where *E*_MXene+*n*Li_ and *E*_MXene_ are the total energies of the MXenes with and without Li-atom respectively, *E*_Li_ is the cohesive energy per Li atom in bulk lithium and *n* is the number of adsorbed Li atoms. The calculated average adsorption energies per Li atom in the first and second layers near saturation are −0.36 eV and −0.02 eV for Ti_2_CS_2_ and −0.59 and −0.14 eV for Ti_2_CN_2_ respectively. Evaluating further Li adsorption beyond two layers reveals that additional Li atoms tend to cluster together, preventing the formation of a stable third layer. Hence the maximum number of adsorbed Li storage ions on the functionalized Ti_2_CS_2_ and Ti_2_CN_2_ 3 × 3 × 1 supercell is 36, which corresponds to a maximum chemical stoichiometry of Ti_2_CS_2_Li_4_ and Ti_2_CN_2_Li_4_ as depicted in Fig. 10(b). Among the three monolayers, Ti_2_CN_2_ exhibits the most negative average adsorption energies over the entire lithiation process, followed by Ti_2_CS_2_ and Ti_2_C, indicating that N surface termination provides the strongest interaction with Li and the highest thermodynamic stability for Li storage.

#### 3.4.2 Lithium storage capacity

A high storage capacity of a metal ion is an important requirement for developing efficient electrode materials for next generation rechargeable metal ion batteries.^91^ The maximum theoretical storage capacity (*C*_m_) of Li-ion battery was calculated using eqn (6).^92^6
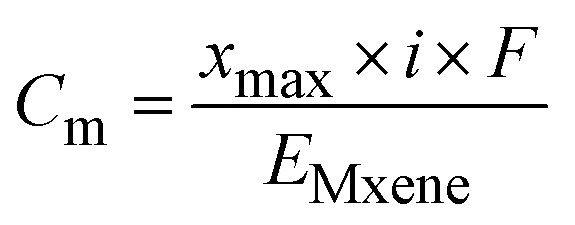
Here *x*_max_ denotes the maximum number of adsorbed lithium ion per formula unit, *i* is the valance state of cations (*i* = 1 for Li), *F* is Faraday's constant (26 801 mAh mol^−1^) and *M*_Mxene_ represents the molar mass (107.75 g per mol for Ti_2_C, 171.87 g per mol for Ti_2_CS_2_ and 135.75 g per mol for Ti_2_CN_2_).

The Li-storage capability of a host material depends on the number of Li atoms it can accommodate, the stability of Li adsorption, and the structural stability of the host. Although Li-host interactions influence adsorption stability and the achievable Li content, the theoretical specific capacity is determined by the number of stored Li atom and the molar mass of the host. Therefore, the capacity difference between Ti_2_CS_2_ and Ti_2_CN_2_ should be discussed in terms of their calculated Li contents and molar masses, while adsorption energies provide additional insight into Li-binding stability.

In the 3 × 3 supercell, pristine Ti_2_C can stably accommodate 18 Li atoms, corresponding to the fully Lithiated composition Li_2_Ti_2_C as shown in Fig. 11(a). Similarly, Ti_2_CS_2_ and Ti_2_CN_2_ can accommodate 18 Lithium atoms at first adsorption layer as shown in Fig. 11(b) and (c). Surface functionalization significantly increases the Li storage capability, allowing both Ti_2_CS_2_ and Ti_2_CN_2_ to accommodate 36 Li atoms through the formation of two stable adsorption layers, giving the compositions Li_4_Ti_2_CS_2_ and Li_4_Ti_2_CN_2_, respectively.

**Fig. 11:**
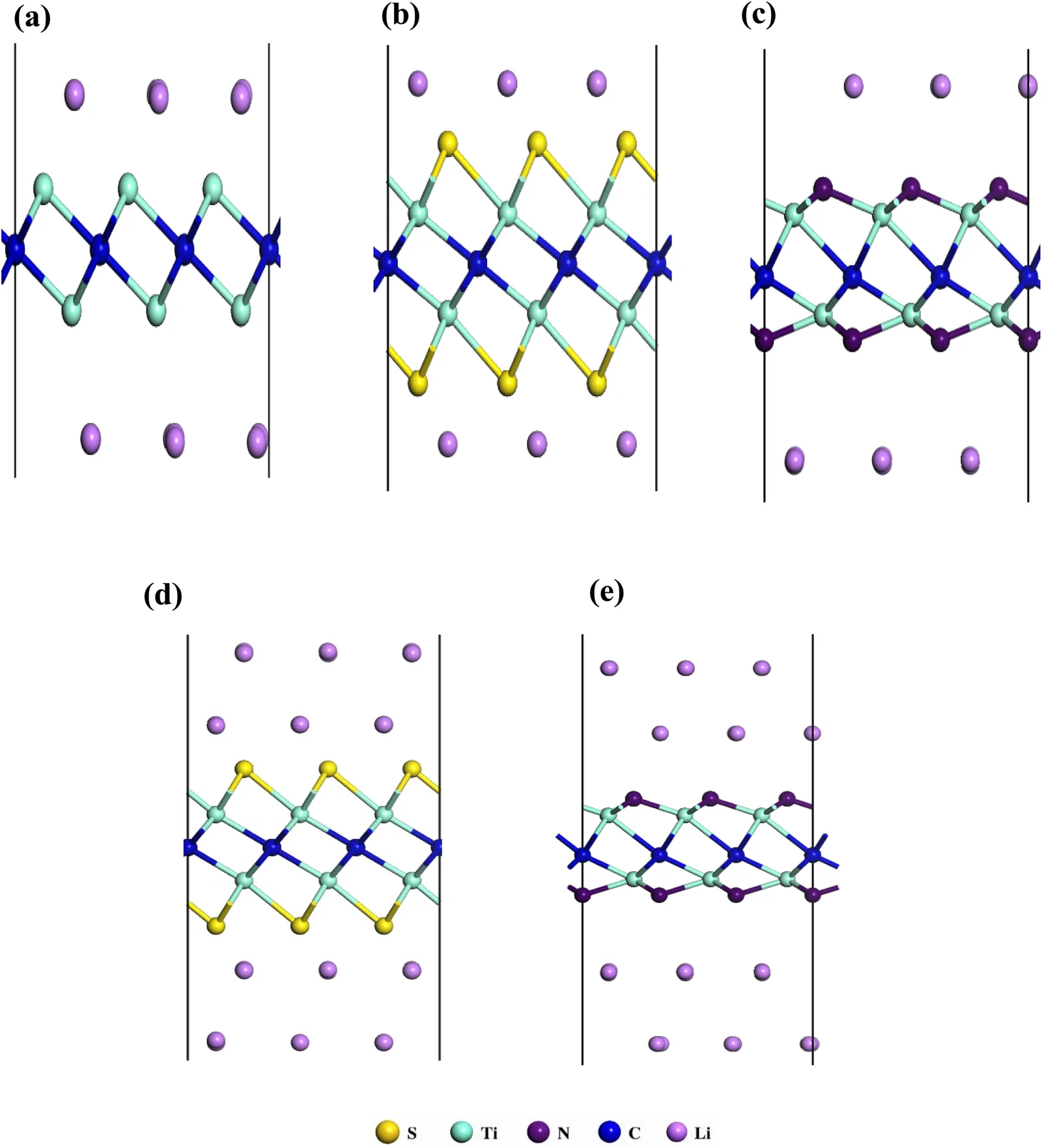
Optimized configurations of Li-intercalated Ti_2_C, Ti_2_CS_2_ and Ti_2_CN_2_ monolayers (a) Li_2_Ti_2_C, (b) Li_2_Ti_2_CS_2_ and (c) Li_2_Ti_2_CN_2_ (first lithiation layer) and (d) Li_4_Ti_2_CS_2_ and (e) Li_4_Ti_2_CN_2_ (fully lithiated state).

To evaluate the thermodynamic stability of the intermediate Li concentrations, formation-energy convex-hull analysis was performed for Li adsorption on Ti_2_CS_2_ and Ti_2_CN_2_, following the approach commonly adopted for two-dimensional electrode materials.^49,93^ The corresponding formation-energy convex-hull plots for Li adsorption on Ti_2_C, Ti_2_CS_2_, and Ti_2_CN_2_ are presented in Fig. 12(a–c), respectively. The concentration states lying on the lower convex hull represent thermodynamically stable configurations relative to the calculated competing compositions, whereas states above the hull are metastable with respect to decomposition into combinations of other compositions. For Ti_2_C, the stable Li concentrations are identified as Li_2_, Li_4_, Li_7_, Li_9_, Li_13_, Li_15_ and L_18_. For Ti_2_CS_2_, the stable concentrations are Li_2_, Li_4_, Li_7_, Li_9_, Li_13_, Li_18_, Li_21_, Li_27_, Li_32_ and Li_36_. For Ti_2_CN_2_, the stable concentrations are Li_3_, Li_6_, Li_9_, Li_15_, Li_24_, Li_28_, Li_32_ and Li_36_.

**Fig. 12:**
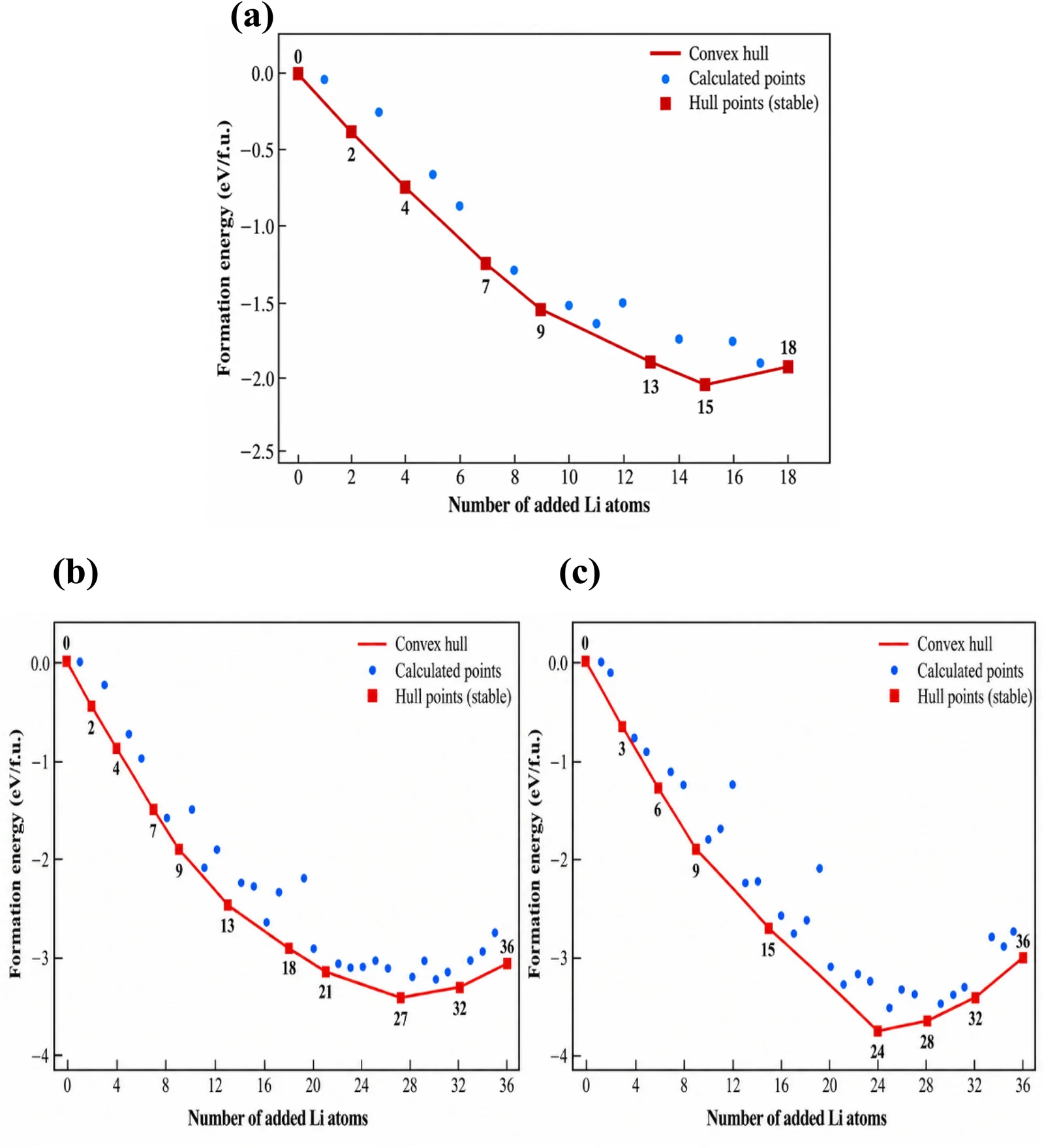
Formation-energy convex-hull plots for Li adsorption on (a) Ti_2_C, (b) Ti_2_CS_2_ and (c) Ti_2_CN_2_.

To assess the structural and thermal stability of the fully lithiated configurations corresponding to the maximum theoretical capacities, AIMD simulations were carried out for Li_4_–Ti_2_CS_2_ and Li_4_–Ti_2_CN_2_ at 500 K for 8 ps. The energy of Li_4_–Ti_2_CS_2_ fluctuates around −524 eV, whereas Li_4_–Ti_2_CN_2_ fluctuates around −544 eV as shown in Fig. 13(a) and (b). Despite these fluctuations, neither system shows a sustained or systematic change in total energy over the 8 ps simulation. The absence of significant structural distortion as in 12(d) and (e) further confirms the structural stability of the fully ion-loaded configurations.

**Fig. 13:**
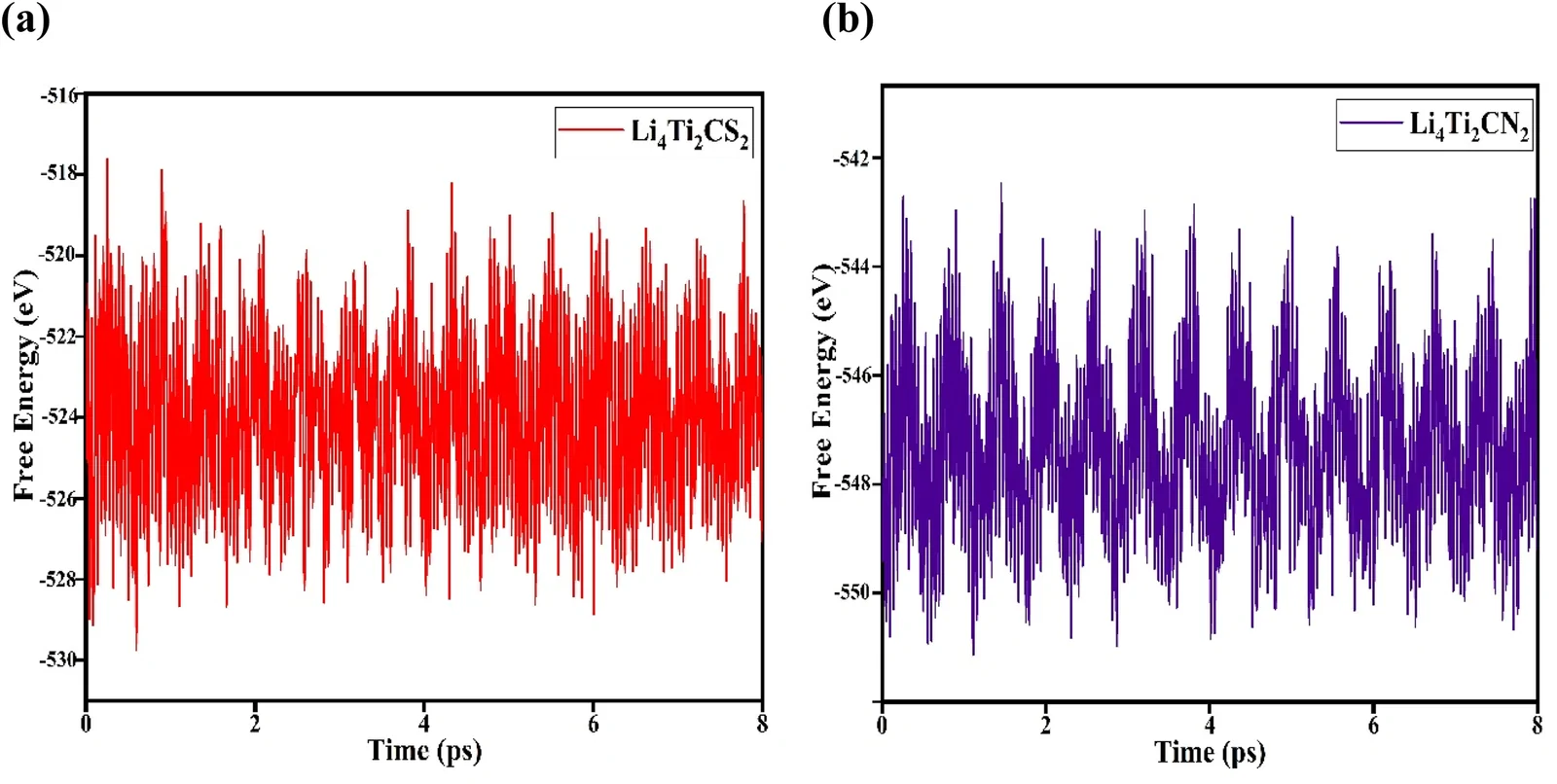
Total-energy evolution of (a) Li_4_–Ti_2_CS_2_ (b) Li_4_–Ti_2_CN_2_ during the 8 ps simulations.

Based on the maximum Li uptake, the calculated specific capacities are 497.5 mAh g^−1^ for Ti_2_C, 623.7 mAh g^−1^ for Ti_2_CS_2_ and 789.7 mAh g^−1^ for Ti_2_CN_2_. As shown in Fig. 14(a), the higher capacities of the functionalized monolayers result from their ability to accommodate an additional stable Li adsorption layer compared with pristine Ti_2_C. Although Ti_2_CS_2_ and Ti_2_CN_2_ adsorb the same maximum number of Li atoms, Ti_2_CN_2_ exhibits the highest gravimetric capacity because of its lower molar mass and stronger interaction with Li, making N-functionalized Ti_2_C the most promising anode among the three materials.

**Fig. 14:**
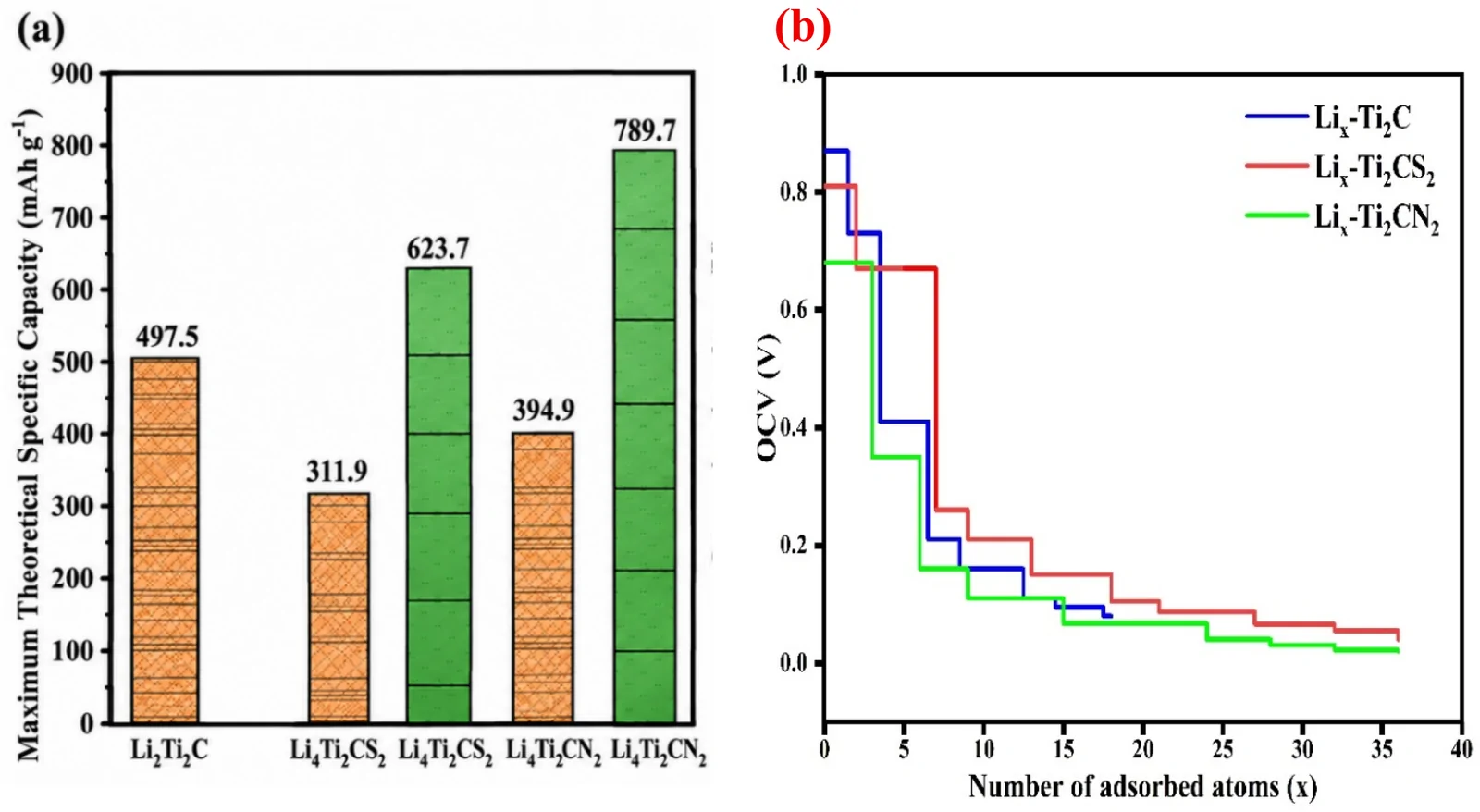
(a) Theoretical Capacity of Li-ion on Ti_2_C monolayer (b) variation of OCV with number of adsorbed Li-ions.

The calculated theoretical specific capacities of the investigated monolayers exceed those reported for several conventional and 2D anode materials such as graphite (∼372 mAh g^−1^),^94^ graphene (338 mAh g^−1^),^95^ and other reported electrodes as Ti_3_C_2_ (320 mAh g^−1^),^96^ TiO_2_ (200 mAh g^−1^),^97^ Mn_2_CF_2_ (167 mAh g^−1^)^98^ and V_2_CSe_2_/Nb_2_CS_*z*_ (592/409-466 mAh g^−1^),^99^ suggesting the strong suitability of all these MXenes for high capacity energy lithium ion battery applications.

#### 3.4.3 Open circuit voltage (OCV)

The OCV serves as an important indicator for determining the overall performance and suitability of electrode materials in metal-ion batteries.^100^ The OCV is calculated using eqn (7).^101^7
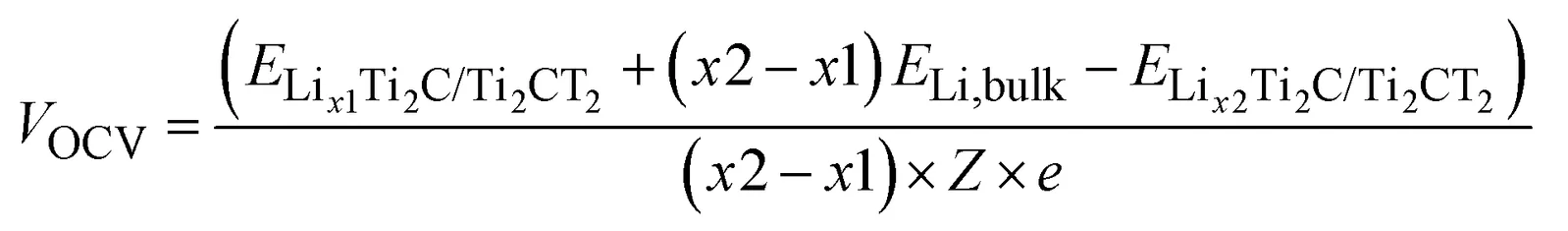
where E_Li_*x*1_Ti_2_C/Ti_2_CT_2__ and *E*_Li_*x*2_Ti_2_C/Ti_2_CT_2__ denotes the total energies of Ti_2_C/Ti_2_CT_2_ with *x*1 and *x*2 adsorbed Li atoms respectively. *E*_Li_ is the energy of a single Li atom in bulk. *x* denotes the number of adsorbed Li-ions, *Z* is charge number and *e* represents the elementary charge of single electron.

The OCV was evaluated to examine the electrochemical behavior of Li insertion into Ti_2_C, Ti_2_CS_2_ and Ti_2_CN_2_ monolayers. As lithiation proceeds, the OCV decreases gradually because the most energetically favorable adsorption sites are occupied first while subsequent Li atoms are accommodated at sites with relatively weaker binding. For pristine Ti_2_C, the voltage decreases from approximately 0.87 V at low Li concentration to 0.08 V at the fully Lithiated composition of Li_2_Ti_2_C (*x*_max_ = 2). A similar trend is observed for the functionalized monolayers, where the OCV decreases from 0.81 to 0.04 V for Li_4_Ti_2_CS_2_ (*x*_max_ = 4) and from 0.68 to 0.02 V for Li_4_Ti_2_CN_2_ (*x*_max_ = 4). The thermodynamically stable concentration states were subsequently considered in determining the OCV profiles and are presented in Fig. 12(b). The observed variations in OCV with increasing Li^+1^ ion adsorption shows a strong dependency on the number of adsorbed Li-ions.

Throughout the lithiation process, all three materials exhibit positive operating voltages within the preferred range (0.1–1V)^102^ for lithium-ion battery anodes, indicating favorable Li insertion without the tendency for metallic Li deposition and it also known that OCVs falling in this range may be advantageous for suppressing lithium dendrite formation and thereby enhancing the overall safety of lithium-ion batteries.^103^ Among the investigated systems, Ti_2_CN_2_ delivers the highest theoretical capacity of 789.7 mAh g^−1^, followed by Ti_2_CS_2_ (623.7 mAh g^−1^) and pristine Ti_2_C (497.5 mAh g^−1^). These capacities exceed that of commercial graphite, while the moderate OCV values suggest that surface functionalization effectively enhances Li storage without compromising electrochemical stability. The combined high storage capacity and suitable voltage characteristics demonstrate that Ti_2_CS_2_ and particularly Ti_2_CN_2_ are promising anode materials for high-performance LIBs.

## 4 Conclusion

In this work, we study the Ti_2_C and functionalized Ti_2_CS_2_ and Ti_2_CN_2_ monolayer using first principles calculations based on DFT calculations ensuring a high density of states at the Fermi level ensuring excellent electronic conductivity for efficient charge discharge cycles. We further investigate their structural stability through phonon dispersion analysis, which shows no imaginary frequencies. AIMD simulations at 500 K confirming robust thermal stability over the simulation period. Surface functionalization with S and N preserves the metallic nature of Ti_2_C, ensuring good electronic conductivity during the lithiation process. Li adsorption is thermodynamically favorable on all investigated monolayers, with Ti_2_CN_2_ exhibiting the strongest interaction, followed by Ti_2_CS_2_ and pristine Ti_2_C. The calculated Li diffusion barriers of 0.24 eV, 0.28 eV, and 0.32 eV for Ti_2_C, Ti_2_CS_2_ and Ti_2_CN_2_, respectively, suggest rapid Li-ion transport and favorable rate capability. Surface functionalization also increases the maximum Li-storage capacity from 497.5 mAh g^−1^ for Ti_2_C to 623.7 mAh g^−1^ for Ti_2_CS_2_ and 789.7 mAh g^−1^ for Ti_2_CN_2_. Furthermore, the calculated OCV values decrease in the order Ti_2_C > Ti_2_CS_2_ > Ti_2_CN_2_, indicating that surface functionalization lowers the OCV relative to pristine Ti_2_C, while the obtained OCV values are within the preferred operating range for lithium-ion battery anodes, indicating reversible Li insertion with good electrochemical stability. Surface functionalization strengthens Li-surface interactions and provides additional favorable sites for Li adsorption, leading to higher Li-storage capacity, but is accompanied by lower OCV values. Among the investigated systems, pristine Ti_2_C shows the most favorable voltage and diffusion characteristics, while Ti_2_CS_2_ and Ti_2_CN_2_ provide stronger Li adsorption and higher storage capacity. Overall, the combined structural stability, metallic conductivity, Li adsorption, diffusion kinetics, storage capacity and voltage characteristics make Ti_2_C-based materials promising candidates for LIB anodes. These findings provide useful theoretical guidance for the development and experimental realization of Ti_2_C-based MXene electrodes.

## Conflicts of interest

The authors declare that we have no conflicts of interest.

## Data Availability

All data supporting the findings of this study are included within the article
